# Supplementary material for: Prevalence of body mass index categories among adults living alone in China: Observational study
Source: PLoS One. 2024 Feb 2;19(2):e0297096. doi: 10.1371/journal.pone.0297096 (PMC10836694; doi:10.1371/journal.pone.0297096)

**Supplemental Appendix to: Prevalence of Body Mass Index Categories Among Adults living alone in China: observational study**

**S1 data source**

Chinese General Social Survey (CGSS) is a comprehensive national survey project jointly conducted by the Department of Sociology at Renmin University of China and the Institute of Sociology at Chinese Academy of Social Sciences. Each round of research uses multi-stage stratified design and data quality controls to ensure the reliability and accuracy of the research results. The sample covers different people and social classes in cities and rural areas across all provinces, autonomous regions, and municipalities in China, thereby having a certain degree of representativeness.

We pooled CGSS data from 2011-2013,2015,2017-2018, and 2021. We harmonized variable definitions across years. There were 73246 participants (those who were live alone or not live alone). Among participants without pregnant, 0.8 % (n = 603) were excluded because of missing data needed to calculate BMI. Less than 0.1% (n = 4) were excluded because of reported height below 80 cm or reported weight below 20 kg. Subsequently, observations with missing values in the primary analysis variables (sex, age) were excluded. After excluding 63,556 non-living alone participants, there were 9,077 living alone adult participants remaining.

We used the CGSS sample weights for the analyses of China. The weighting scheme for each final survey unit is determined by the product of the initial design weight assigned to each household and the adjustment weight calculated for the execution status, resulting in a refined estimate of the underlying population parameters.

The CGSS collected demographic and socioeconomic information, including age, gender, ethnic, educational level, physical activity, health insurance, and family annual income. Education levels are classified by the distribution of the variable (Specific survey questions for the variables are listed in supplement table 1). For annual income, CGSS used total income from various sources.

References:
Chinese General Social Survey. CGSS Sampling Design. Available at: http://cgss.ruc.edu.cn/xmwd/cysj.htm

**S1 Table Specific survey questions for all the variables in CGSS**

| Variables | CGSS Questions |
| --- | --- |
| Live alone | How many people are currently living together in your household, including yourself? |
| Education level | Your current highest level of education is (including current education) |
| Health insurance | May I ask what kind of medical insurance you currently enjoy |
| Annual income | What is your total household income |
| Cardiovascular disease | None |
| Hypertension | None |
| Physical activity | In the past year, have you regularly engaged in the following activities in your free time - taking part in physical exercise |
| Diabetes | None |
| Smoke status | None |

| Characteristics | Obesity | Overweight |
| --- | --- | --- |
| Male | 7.9(7.1,8.7) | 33.3(31.9,34.7) |
| Female | 8.1(7.3,8.9) | 29.4(28.1,30.7) |
| 18-24y | 5(2.9,7.1) | 10.6(7.6,13.5) |
| 25-34y | 5.7(3.8,7.7) | 22(18.5,25.5) |
| 35-44y | 7.4(4.9,10) | 35.9(31.2,40.6) |
| 45-54y | 9.4(7.4,11.4) | 38.3(34.9,41.8) |
| 55-64y | 7.5(5.8,9.3) | 36(32.7,39.3) |
| >64y | 6.8(5.5,8.2) | 28.7(26.3,31.1) |
| Physical activity | 7.7(6.5,8.8) | 31.5(29.5,33.4) |
| Health insurance | 7(6.3,7.8) | 30.2(28.8,31.5) |
| <¥20000 | 5.9(4.8,7.1) | 26.9(24.8,29.1) |
| ¥20000-¥40000 | 7.9(6.3,9.4) | 33.6(30.8,36.3) |
| ¥40000-¥75000 | 8.3(6.5,10.1) | 31.4(28.4,34.4) |
| >¥75000 | 8.2(5.9,10.4) | 29.5(25.9,33.1) |
| Primary school | 7.1(5.8,8.4) | 26.7(24.6,28.8) |
| Up to high school | 7.7(6.4,8.9) | 32.1(29.9,34.3) |
| College graduate | 6.3(4.2,8.3) | 33.3(29.2,37.4) |

**S2 Table Prevalence of BMI categories among Chinese adults with Probit regression**

**S1 Figure The interaction between age and gender among adults living alone**


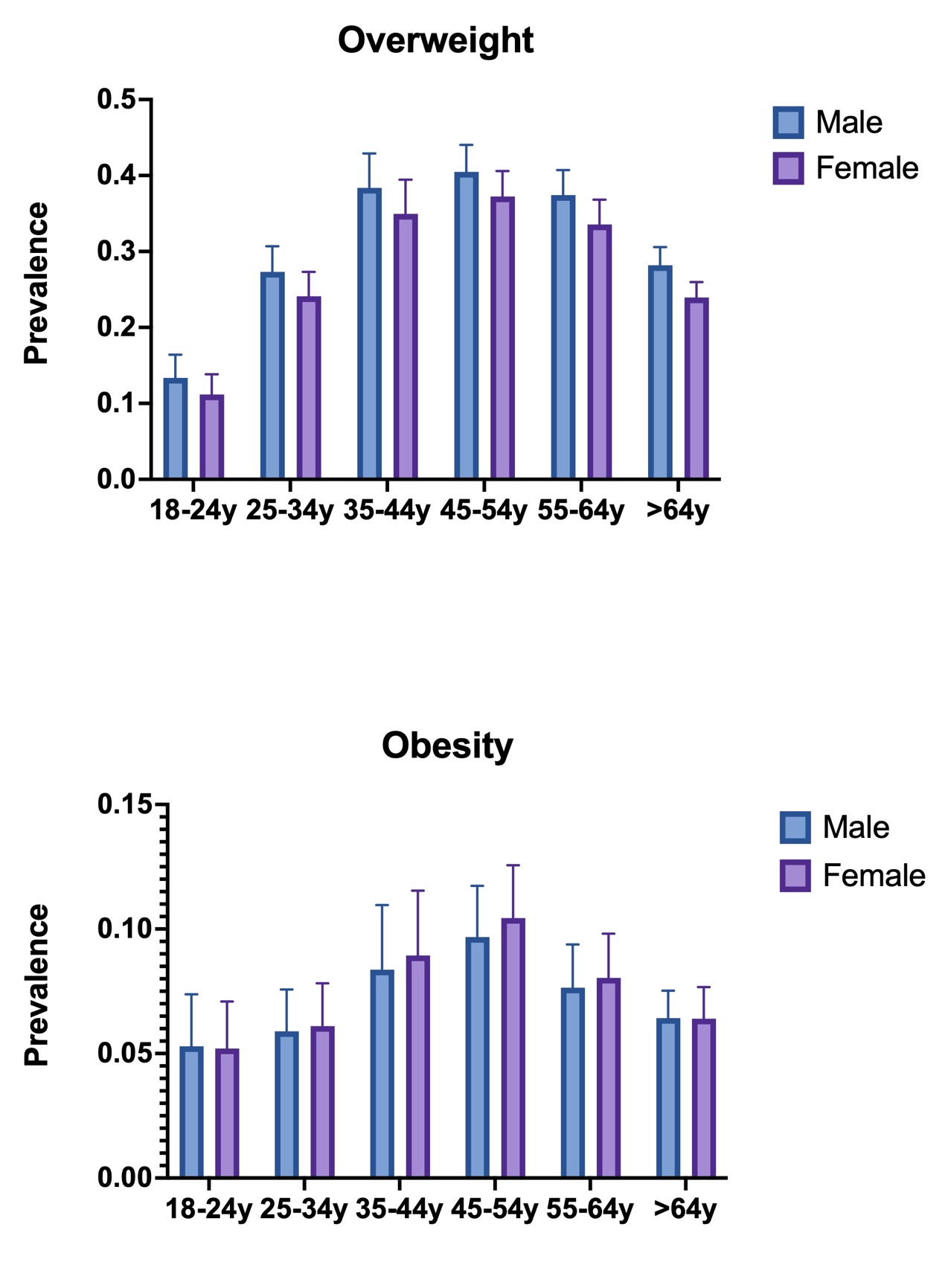


**S2 Figure Prevalence of BMI Categories in Solitary Populations in Urban and Rural Areas**


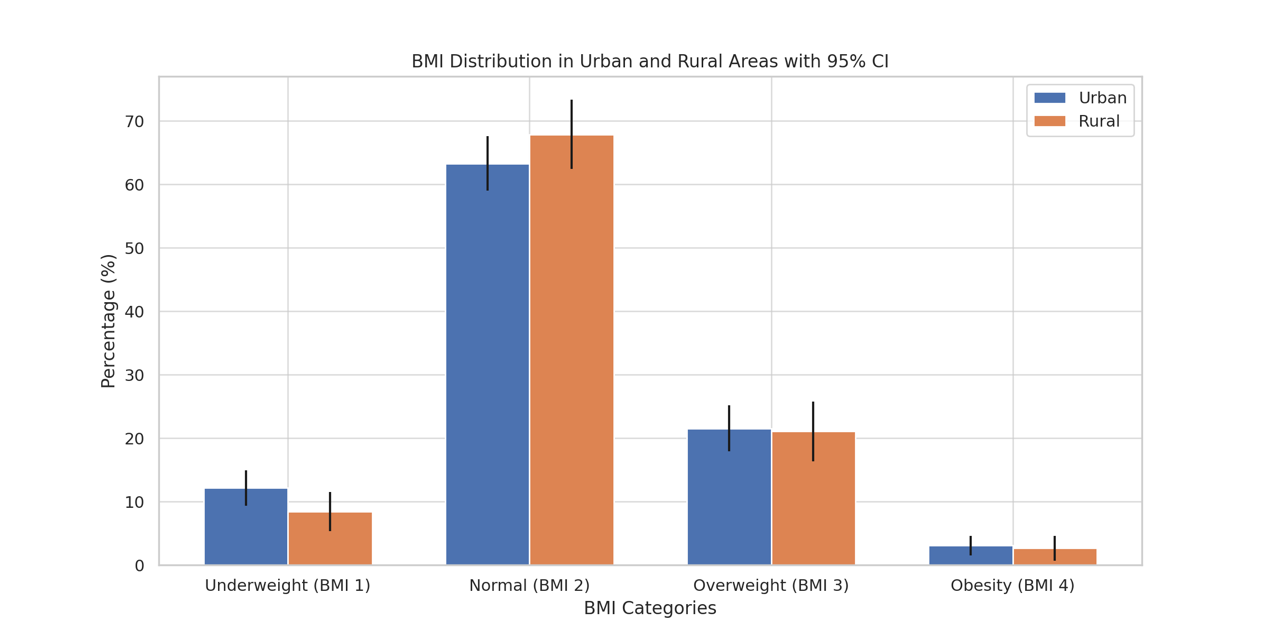

Supplement: S1 File — (DOCX) [file pone.0297096.s001.docx]
