# Supplementary material for: Prevalence of body mass index categories among adults living alone in China: Observational study
Source: PLoS One. 2024 Feb 2;19(2):e0297096. doi: 10.1371/journal.pone.0297096 (PMC10836694; doi:10.1371/journal.pone.0297096)
Supplement: S3 File — (PDF) [file pone.0297096.s003.pdf]

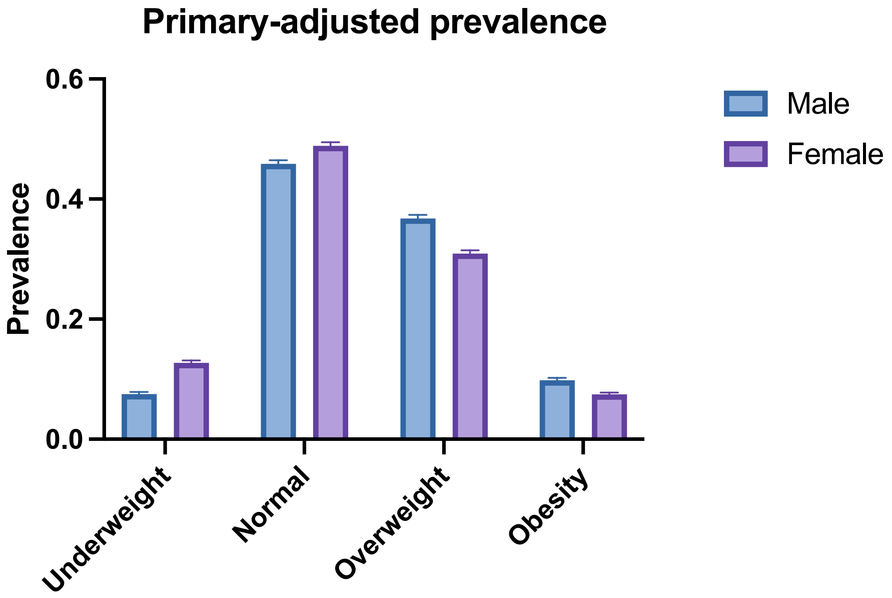
Primary-adjusted prevalence
Prevalence
0.0
0.2
0.4
0.6
Underweight
Normal
Overweight
Obesity
Male
Female

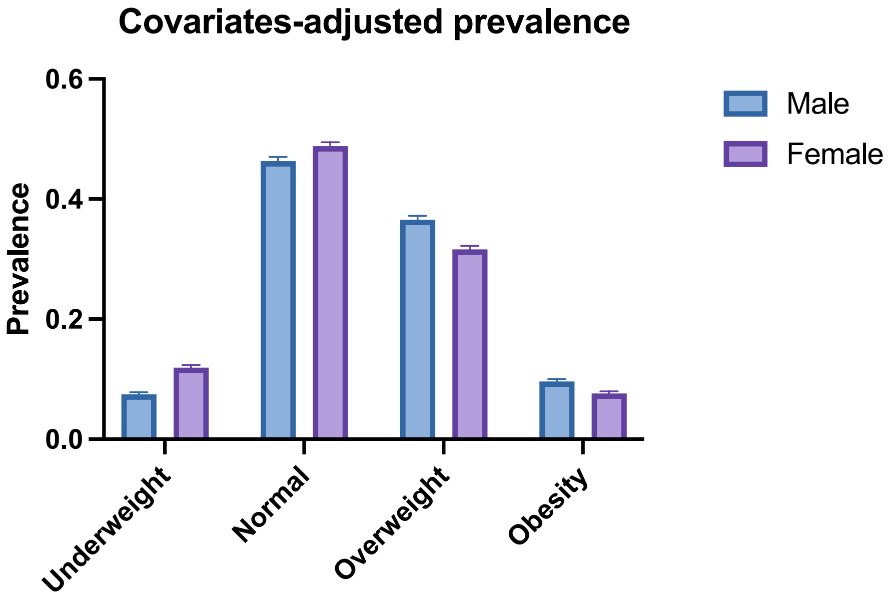
Covariates-adjusted prevalence
Prevalence
0.0
0.2
0.4
0.6
Underweight
Normal
Overweight
Obesity
Male
Female

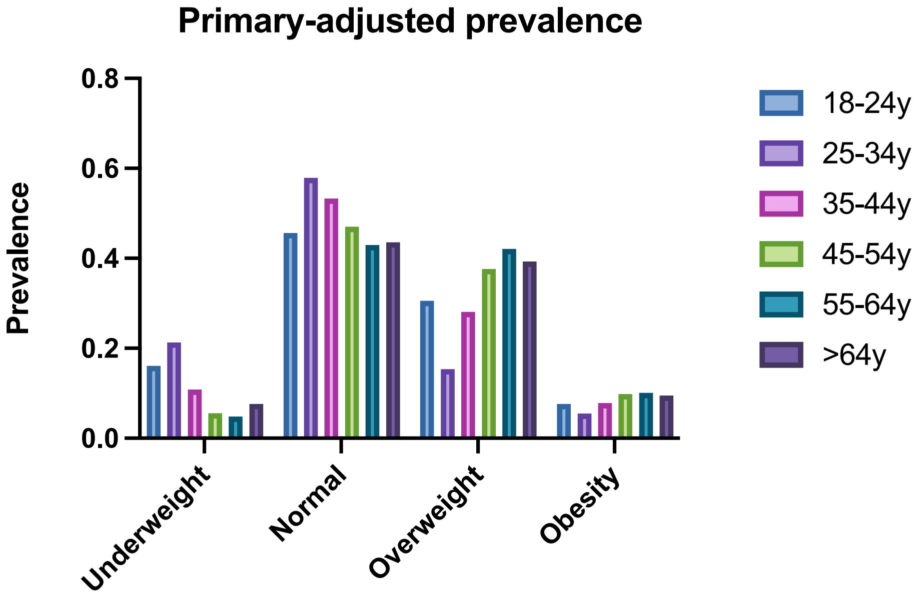
Primary-adjusted prevalence
Prevalence
0.0
0.2
0.4
0.6
0.8
Underweight
Normal
Overweight
Obesity
18-24y
25-34y
35-44y
45-54y
55-64y
>64y

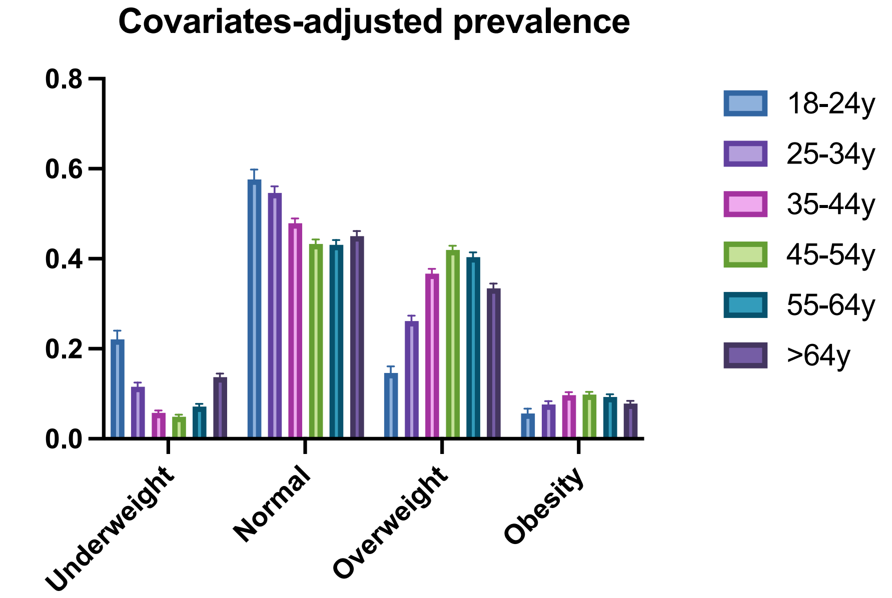
Covariates-adjusted prevalence
0.0
0.2
0.4
0.6
0.8
Underweight
Normal
Overweight
Obesity
18-24y
25-34y
35-44y
45-54y
55-64y
>64y
