# Supplementary figures and images for: Prevalence of body mass index categories among adults living alone in China: Observational study
Source: PLoS One. 2024 Feb 2;19(2):e0297096. doi: 10.1371/journal.pone.0297096 (PMC10836694; doi:10.1371/journal.pone.0297096)

A

Underweight

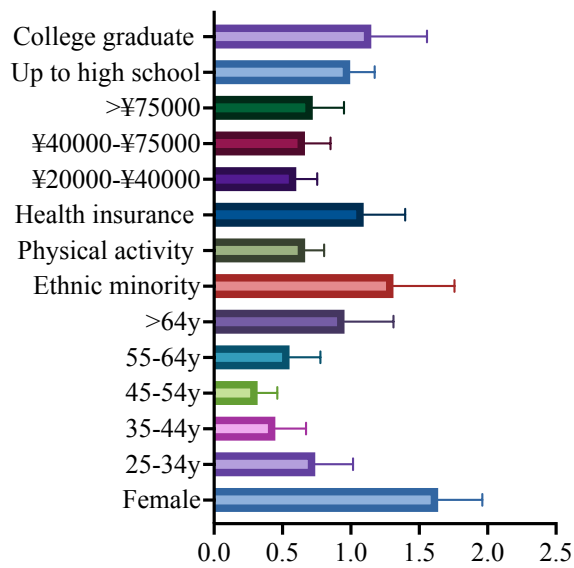

B

Overweight

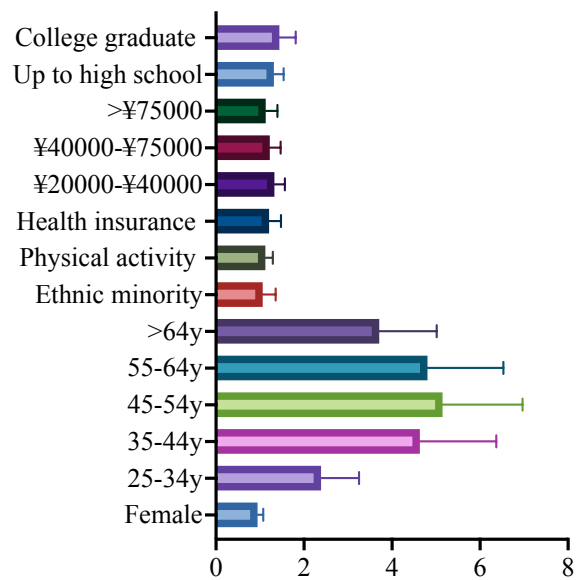

C

Obesity

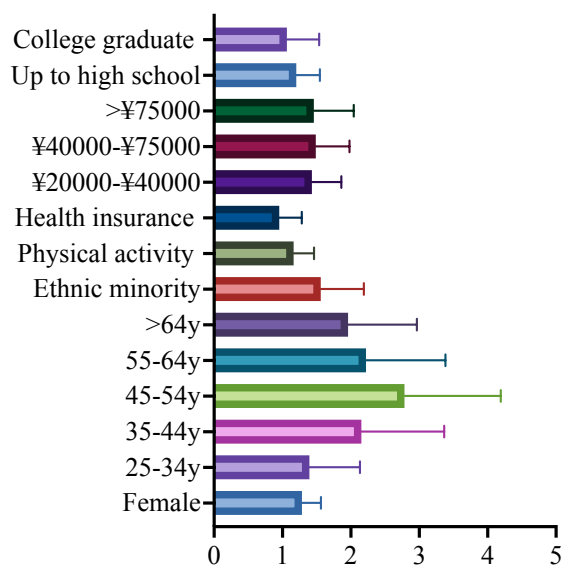

D

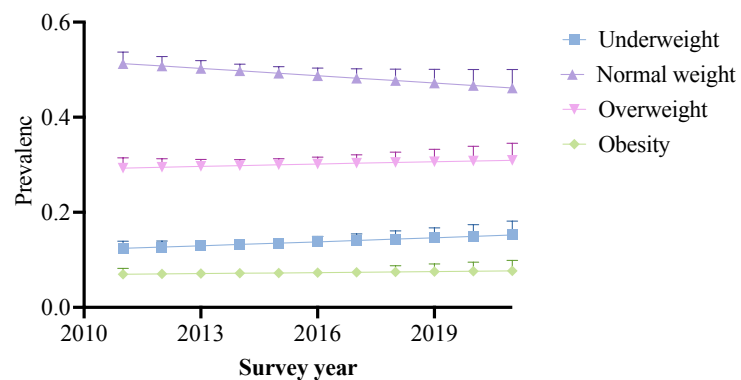

Supplement: S4 File — (PDF) [file pone.0297096.s004.pdf]
