# Supplementary material for: Prevalence of body mass index categories among adults living alone in China: Observational study
Source: PLoS One. 2024 Feb 2;19(2):e0297096. doi: 10.1371/journal.pone.0297096 (PMC10836694; doi:10.1371/journal.pone.0297096)
Supplement: Attachment — Submitted filename: Response to Reviewers.docx [file pone.0297096.s005.docx]

Response to the reviewer comments for PONE-D-23-14813, "***Prevalence of Body Mass Index Categories Among Adults living alone in China: Observational Study***"

I would like to thank the Editor(s) and the reviewers for their constructive comments and questions on our manuscript. I believe addressing these has helped us improve the paper substantially. Please find below a response to the specific points raised by the reviewers.

**Editor Comments:**

**The paper reads well in general. I suggest that the authors look into different modeling strategies, e.g., Yen et al. (2009) that used switching regression with known regime.**

**There are some minor editorial/cosmetic issues. For example, LA needs to be defined first -- it appears to be living alone from the context -- and used consistently.**

**Yen ST, Chen Z, Eastwood DB. (2009) Lifestyle and obesity: a multi-regime switching regression analysis with an endogenous regressor. Health Services Research, 44:1345-1369.**

**Response:**

Thank you for your valuable feedback. We appreciate your suggestion to explore different modeling strategies, such as the one used by Yen et al. (2009) involving switching regression with known regimes. However, due to the specific application domain, data constraints, and the inherent uncertainties surrounding the regime, we opted for an alternative modeling approach, namely Probit regression, as a substitute for logistic regression. Additionally, we have addressed the concern regarding the definition of 'LA' by providing a clear definition for it in the introduction section of the paper. The changes have been marked in red.

**Response:**

**Comments from the external peer reviewers**

**1. The introduction provides relevant background but contains some repetitive details. Try to synthesize and condense the background concisely to highlight gaps this study aims to address.**

**Response:** Thank you for your feedback. We have revised the introduction section to condense the background information and eliminate repetitive details. The revised introduction now provides a more concise overview of the relevant background while emphasizing the specific gaps that our study aims to address. The changes have been marked in green.

**2. Should improve the graph quality of the results tables and figure**

**Response:** Thank you for your feedback. We have made the necessary improvements to enhance the quality of the results tables and figures, with particular attention to Figure 2.

**3. Carefully proofread the entire manuscript to improve grammar, wording, and readability issues.**

**Response:** Thank you for your suggestion. We have carefully proofread the entire manuscript to address any grammar, wording, and readability issues.

**4. The methods should explain the CGSS sampling scheme and representativeness more clearly. Discuss any limitations of self-reported data.**

**Response:** Thank you for your valuable feedback. We have included detailed information about the CGSS sampling scheme and its representativeness in the supplementary materials. Additionally, we have discussed the limitations of self-reported data in the discussion section of the manuscript. The changes have been marked in green.

**5. Include p-values alongside the 95% CIs for ORs in tables/figures.**

**Response:** Thank you for your suggestion. As all the p-values are less than 0.001, we did not include them in the main body of the tables/figures. However, we have now added the p-values in methods section for clarity.

**6. Use consistent formatting for income categories in Table 1 versus Figure 2.**

**Response:** Thank you for pointing out the formatting inconsistency in the income categories between Table 1 and Figure 2. We will ensure that the formatting for income categories is made consistent across both the table and the figure in the revised version."

**7. Could examine interactions between key variables like gender and age.**

**Response:** Thank you for your suggestion. We have added the analysis of interactions between age and gender in S1 Figure.

8. The discussion could articulate the implications and importance of the findings more clearly, especially for public health practice and policymaking. Cite the following papers to enrich the discussion and background

**Response:** Thank you for your input. The implications of our findings for public health and policymaking have been clarified in the discussion. The suggested papers have also been included to enrich both the background and discussion sections.
