# Supplementary material for: Prevalence of body mass index categories among adults living alone in China: Observational study
Source: PLoS One. 2024 Feb 2;19(2):e0297096. doi: 10.1371/journal.pone.0297096 (PMC10836694; doi:10.1371/journal.pone.0297096)
Supplement: Attachment — Submitted filename: plosoneBMI.docx [file pone.0297096.s006.docx]

**Dear Editor,**

Thank you for considering me as a reviewer for this publication in your esteemed journal

**PLOS ONE.** I have provided my comments as follows:

- Some research suggests that other measures of body fat, such as skinfold thicknesses, bioelectrical impedance, underwater weighing, and dual energy x-ray absorption, may be more accurate than BMI. The waist circumference (sometimes divided by height) is also a simple measure of fat distribution. Of course, checking waist circumference is an easy and less expensive method that can help enrich the results of this research. Therefore, it is suggested to check and add waist circumference and its relationship with BMI.
- It is suggested to compare BMI in different regions of China (urban and rural).
- It is suggested that the results of this study be compared with the results in other countries of this region.
- • As you know, some factors such as psychosocial status, genetic factors and physical health can affect BMI. Please add explanations about these factors in the discussion.
- Has the relationship between the type of diet (daily calorie intake) and BMI been investigated? Please explain.
