# Supplementary material for: Prevalence of body mass index categories among adults living alone in China: Observational study
Source: PLoS One. 2024 Feb 2;19(2):e0297096. doi: 10.1371/journal.pone.0297096 (PMC10836694; doi:10.1371/journal.pone.0297096)
Supplement: Attachment — Submitted filename: Response V1.docx [file pone.0297096.s007.docx]

Response to the reviewer comments for PONE-D-23-14813R1, "***Prevalence of Body Mass Index Categories Among Adults living alone in China: Observational Study***"

I would like to thank the reviewers for their constructive comments and questions on our manuscript. I believe addressing these has helped us improve the paper substantially. Please find below a response to the specific points raised by the reviewers.

**Journal Requirements:

Please review your reference list to ensure that it is complete and correct. If you have cited papers that have been retracted, please include the rationale for doing so in the manuscript text, or remove these references and replace them with relevant current references. Any changes to the reference list should be mentioned in the rebuttal letter that accompanies your revised manuscript. If you need to cite a retracted article, indicate the article’s retracted status in the References list and also include a citation and full reference for the retraction notice.**

**Response:** Thank you very much for your guidance. We have reviewed all the references to ensure their appropriateness and made modifications to reference number 14. We have also added red markings in the manuscript to indicate these changes, as per your request.

**Comments from the external peer reviewers**

**Reviewer #1**

**The authors have addressed majority questions raised by the Editor and reviewers. Figures still need higher resolution and legends.**

**Response:** We greatly appreciate your valuable feedback on our manuscript. Regarding the issue you mentioned regarding image resolution, we would like to clarify that the images we uploaded initially met the required resolution. However, the problem arose during the conversion of these images into PDF files, which resulted in a loss of clarity in the final PDF.

To address this concern, we have taken the necessary steps to reprocess all the images and have uploaded them in PDF format. We hope this improvement will meet your expectations and provide higher clarity and quality for the images in the final version.

**Reviewer #3**

**1. Some research suggests that other measures of body fat, such as skinfold thicknesses, bioelectrical impedance, underwater weighing, and dual energy x-ray absorption, may be more accurate than BMI. The waist circumference (sometimes divided by height) is also a simple measure of fat distribution. Of course, checking waist circumference is an easy and less expensive method that can help enrich the results of this research. Therefore, it is suggested to check and add waist circumference and its relationship with BMI.**

**Response:** Thank you very much for your feedback and suggestions. Regarding the use of CGSS data, it is indeed true that we do not have waist circumference information available in the dataset for analysis. Therefore, we are unable to include waist circumference data and its relationship with BMI in this study. In our research, we have focused on analyzing the available information in the CGSS data to explore the relationship between BMI and other factors.

**2. It is suggested to compare BMI in different regions of China (urban and rural).**

**Response:** Thank you for your valuable feedback. We have already conducted a comparison of BMI in different regions of China (urban and rural) in the manuscript. The relevant text has been emphasized in red for clarity, and the corresponding figures have been included in the supplementary materials. We hope these adjustments address your suggestion.

**3. It is suggested that the results of this study be compared with the results in other countries of this region.**

**Response:** Thank you for your suggestion. We have indeed included a comparison of the results of this study with those from other countries in the same region in the discussion section of the manuscript. We have emphasized this comparison using red font to highlight its significance. We hope that this addresses your recommendation.

**4. As you know, some factors such as psychosocial status, genetic factors and physical health can affect BMI. Please add explanations about these factors in the discussion.**

**Response:** Thank you for your feedback. We appreciate your suggestion, and we have already incorporated explanations regarding the factors that can affect BMI, such as psychosocial status, genetic factors, and physical health, in the discussion section of the manuscript. The additional content has been marked in red. We hope that these additions enhance the comprehensiveness of our study.

**5. Has the relationship between the type of diet (daily calorie intake) and BMI been investigated? Please explain.**

**Response:** Thank you for your inquiry. We did not investigate the relationship between diet type (daily calorie intake) and BMI in our study because we did not have access to relevant data for this specific aspect. However, we have acknowledged the importance of diet as a potential factor influencing BMI and have discussed it in the context of existing research. We have also provided references to other studies that have explored this relationship in more detail. The additional content has been marked in red.
